# Esophageal Granular Cell Tumor: A Case Report and Review of Literature

**DOI:** 10.7759/cureus.782

**Published:** 2016-09-14

**Authors:** Nishitha Thumallapally, Uroosa Ibrahim, Mayurathan Kesavan, Qing Chang, Lynne Opitz, Meekoo Dhar, Sherif Andrawes

**Affiliations:** 1 Internal Medicine, Staten Island University Hospital; 2 Department of Hematology/Oncology, Staten Island University Hospital; 3 Department of Gastroenterology, Staten Island University Hospital; 4 Department of Pathology, Staten Island University Hospital

**Keywords:** esophagus, granular cell tumors, endoscopy, submucosal resection

## Abstract

Granular cell tumors (GCTs) are soft tissue neoplasms that originate from Schwann cells. They occur predominantly in the oral cavity, skin, and breast tissues. Gastrointestinal GCTs are very rare, accounting for only eight percent of all GCTs, most of which are located in the esophagus. Endoscopic ultrasound has been a breakthrough in diagnosing GCTs because it provides precise information on the depth of tumor invasion, thus narrowing the differential diagnosis of subepithelial lesions in the esophagus. However, the definitive diagnosis requires histological confirmation of the lesion. Here, we report a case of esophageal GCT that was identified incidentally and removed by endoscopic mucosal resection.

## Introduction

Granular cell tumors (GCTs) are uncommon, benign soft tissue neoplasms originating in the Schwann cells of the nerve sheath. They were first reported in 1920 by Abrikossoff in a case series composed of benign tumors that were removed from the tongue [1]. Initially, GCTs were believed to occur in skeletal muscles only. However, subsequent studies revealed that these tumors can manifest at any site in the body, with a predilection towards the skin, the oral cavity, and breast tissue [1]. GCTs involving the gastrointestinal tract are relatively rare, accounting for only about eight percent of all GCTs, among which esophageal involvement is seen only in two percent [2]. Since 1930, about 250 cases of esophageal GCTs have been reported in the literature. Owing to its rarity and low incidence, there is presently no consensus regarding appropriate esophageal GCT diagnostic testing, management, and surveillance. In this work, we take the opportunity to describe a case of esophageal GCT that was found fortuitously and subsequently removed en block by endoscopic mucosal resection (EMR).

## Case presentation

**Figure 1:**
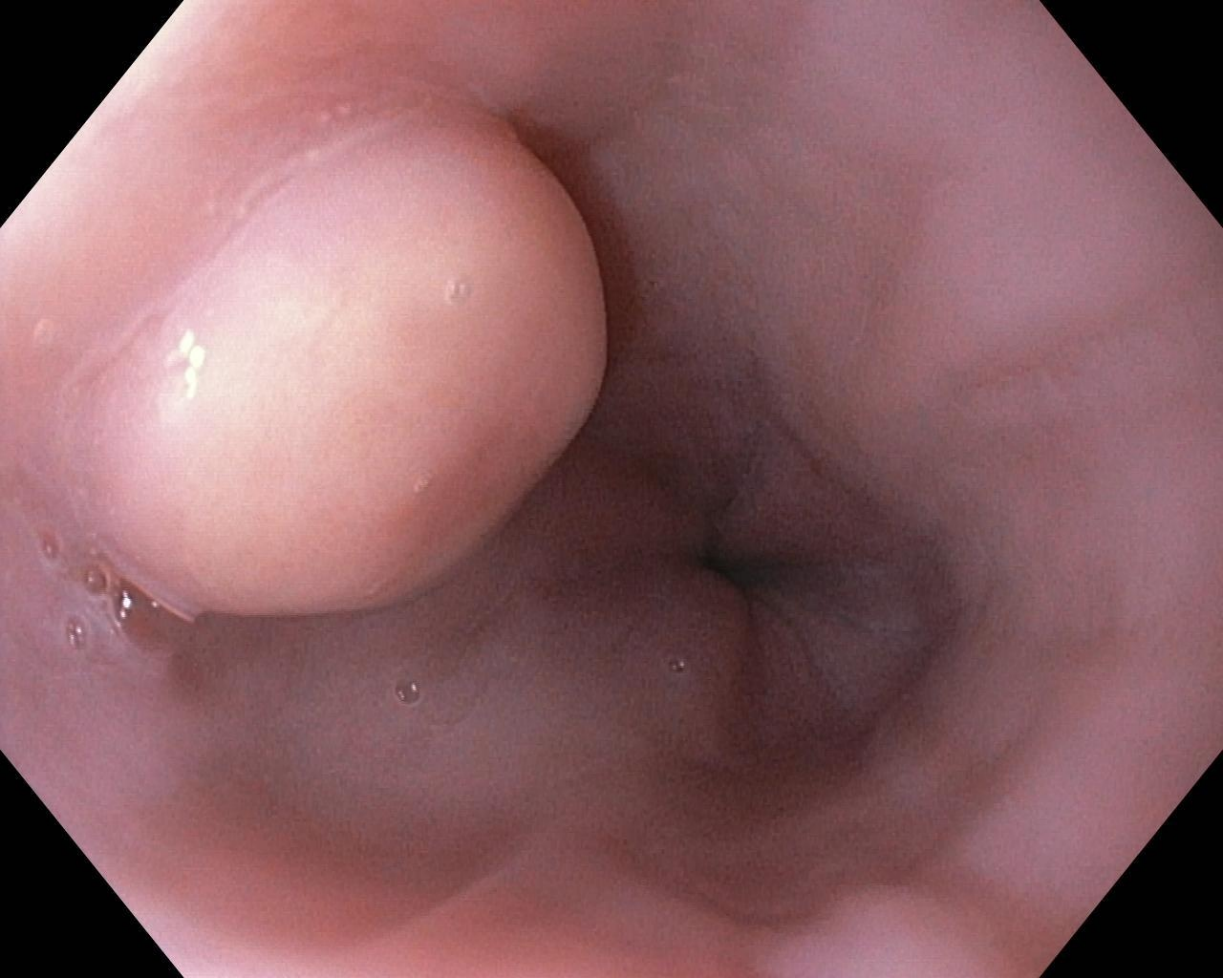
Subepithelial lesion in the distal esophagus 30 cm from the oral cavity.

A 19-year-old morbidly obese Caucasian woman was given preoperative esophagogastroduodenoscopy before bariatric surgery. During endoscopy, she was found to have a one centimeter subepithelial, white nodular lesion in the distal part of the esophagus, 30 cm from the oral cavity, which was further evaluated by endoscopic ultrasound (EUS; see Figure 1).

**Figure 2:**
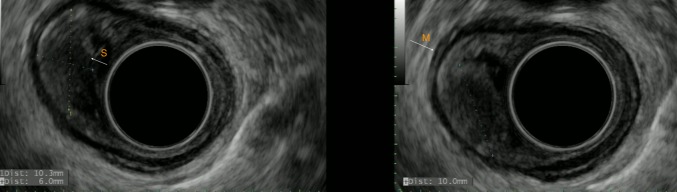
Endoscopic ultrasonography images revealing a well-demarcated, hypoechoic, homogenous lesion arising from the submucosal layer as depicted by white arrow (S). M represents muscularis propria.

Further evaluation using EUS confirmed the presence of a 10.3 x 6 mm well-demarcated hypoechoic lesion confined to the submucosa without invasion into the muscularis propria (Figure 2).

**Figure 3:**
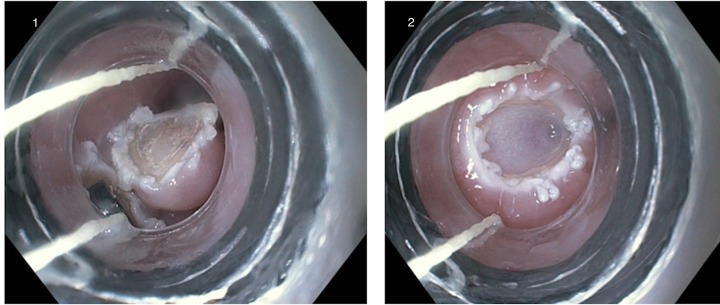
1) Cap-assisted endoscopic mucosal resection of the lesion 2) Mucosal defect after EMR.

The lesion was successfully resected en bloc by a cap-assisted EMR technique (Figure 3).

**Figure 4:**
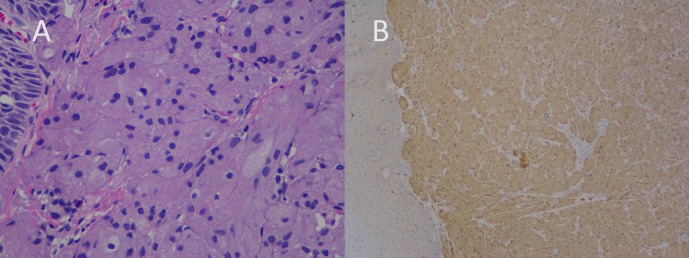
Histopathology revealed (A) the presence of a large round/polygonal cells with abundant cytoplasm and small uniform nuclei, and (B) IHC staining for S-100 was positive.

The mucosal defect was subsequently closed with three endoclips. Careful observation of the excised specimen confirmed the complete removal of the lesion. The cross-section was composed of pink polypoid tissue measuring 0.9 × 0.8 × 0.5 cm in volume. A microscopic analysis revealed large polygonal cells with abundant granular cytoplasm. Cytoplasm, as well as the cell nuclei, showed diffuse Periodic Acid Schiff (PAS) positivity. The immunohistochemical (IHC) analysis exhibited strong reactivity for S-100 protein, thus confirming the GCT diagnosis (Figure 4).

Informed consent has been obtained from the patient. No patient-identifying information is disclosed in this paper.

## Discussion

GCTs are uncommon benign tumors originating in primitive nerve cells. Esophageal GCTs are particularly rare, with very few cases reported in the extant literature. However, owing to the introduction of EUS and advances in endoscopic resection, these tumors are presently diagnosed more easily.

Though esophageal GCTs can occur at any age, they are most prevalent in the 40- to 60-year-old cohort. Moreover, a striking female and African-American predominance, when compared to male and Caucasian patients, respectively, has been noted [3]. Authors of existing studies report that esophageal GCTs predominantly occur in the distal esophagus. In a study conducted by Orlowsa, et al. 65% of esophageal GCTs were seen in the distal part of the esophagus, while the remaining 20% and 15% were found in the middle and proximal part, respectively [4]. Though commonly reported as solitary lesions, esophageal GCTs can occur at multiple sites synchronously or metachronously. According to Orlowsa, et al. up to 11% of patients may have two or more esophageal GCTs [4]. Despite several reports suggesting the presence of multifocal GCTs in the gastrointestinal tract, patients are not typically subjected to a complete endoscopic examination of both the upper and lower gastrointestinal tract. As esophageal GCTs are usually asymptomatic, the majority of lesions are found incidentally during the investigation for other problems. Multifocal and large (i.e. > 1 cm) tumors may present with symptoms such as dysphagia and acid reflux.

Typically, the most striking endoscopic feature of esophageal GCT is the presence of a sessile, white-to-grey elevated lesion with a smooth surface. In rare cases, affected patients may present with ulceration and necrosis. Although GCTs are usually located within the submucosa, these tumors can involve the mucosa and muscularis propria. In particular, when the muscularis propria is involved, differentiating GCTs from leiomyoma—the most common benign intramural tumor of esophagus—becomes difficult.

EUS remains the primary GCT diagnostic tool, as it is the only currently available technique that allows for ascertaining the depth of tumor invasion and at the same time assists in the sampling of the lesion by fine needle aspiration (FNA). The study conducted by Palazzo, et al. is a valuable source for understanding the EUS features of esophageal GCTs [5]. The authors studied 15 patients with biopsy-proven GCTs and, in their published work, reported the EUS patterns they had observed. The key characteristics described were tumor size of lesser than 2 cm with hypoechoic and homogeneous pattern arising in the mucosa or submucosa. In contrast to general characteristics, GCTs can also present as hyperechoic lesions, often leading doctors to misdiagnosing them as lipomas [6].

Though EUS helps in narrowing the differential diagnosis of subepithelial lesions, the definitive diagnosis is always based on microscopic examination of the specimen. Nests of polygonal cells with small, round nuclei that are rich with granular cytoplasm are among the key histopathologic features of GCT, often accompanied in pseudoepitheliomatous hyperplasia in the overlying mucosal epithelium. When there are confounding features histologically, IHC analysis is very helpful. The use of positive staining with S-100 was first reported in 1986. In subsequent years, other authors used PAS, nestin, vimentin, and other markers [7]. Negative markers include desmin, CD117, CD34, fibronectin, and carcinoembryonic antigen. Rarely, GCTs can be misdiagnosed as esophageal squamous cell cancers (spindle cell carcinomas in particular) when deep granular cells in a specimen with pseudoepitheliomatous hyperplasia are not observed. Although esophageal GCTs are generally benign, malignant degeneration has been reported in one to 2 % of the cases, especially in extremely aggressive tumors that exceed 15 mm in length. The key characteristic features that differentiate malignant from benign GCT are the presence of nuclear fission, necrosis, a high mitotic index, and nuclear-cytoplasmic ratios in malignant lesions [8].

Presently, GCTs can be managed by (a) conservative endoscopic follow-up of patients with tumors < 1 cm in length, provided that biopsy does not reveal any signs of malignancy; (b) removal of lesions of > 2 cm in length by EMR or endoscopic submucosal dissection (ESD); or (c) resecting lesions that are symptomatic or displaying malignant features. Though EMR is the most widely used method for resecting GCTs, its limitations should be noted. In tumors exceeding 1 cm in size, removal may not always be complete. Also, when the tumor invades deeper layers, muscularis propria in particular, it is difficult to achieve a complete resection. According to the report issued by Mayo Clinic, esophageal GCT was unsuccessful when EMR was used in the removal of a 13-mm esophageal GCT [9]. Thus, ESD has emerged as a promising approach in such contexts. In this technique, the mucosa surrounding the tumor tissue is removed circumferentially, along with complete dissection of the submucosal layer underneath the lesion. ESD is clearly advantageous in patients presenting with larger lesions (> 15 mm), as it allows complete histologic resection and removal in one piece [10]. The drawbacks of this procedure include a longer duration, greater risk of bleeding and perforation when compared to EMR. Surgery should be considered in malignancy, lesions involving deeper layers that are difficult to remove through EMR or ESD, and when there are contraindications for ESD/EMR. Empirical evidence suggests that the highest incidence of complications is associated with surgery including the laparoscopic approach, which also requires a longer recovery period. Treatment decisions with respect to surgical intervention versus endoscopic resection should always be based on the characteristics of the tumor in each individual and the physician’s expertise. Given the potential for malignant degeneration and recurrence, patients are often advised to undergo long-term follow-up with endoscopic surveillance. 

## Conclusions

Esophageal GCTs should be considered when presenting with subepithelial lesions in the distal part of the esophagus in asymptomatic patients. EUS is the most beneficial diagnostic tool, which helps in planning appropriate treatment based on the depth of invasion. Complete endoscopic resection of esophageal GCTs either by EMR or ESD is recommended. Long-term endoscopic surveillance is always advised due to the high rate of recurrence.
